# The Plastic treaty: What is in it for Africa?

**DOI:** 10.1002/puh2.83

**Published:** 2023-04-04

**Authors:** Deborah Oluwaseun Shomuyiwa, Francisca Ogochukwu Onukansi, Maria Ivanova, Don Eliseo Lucero‐Prisno

**Affiliations:** ^1^ Faculty of Pharmacy University of Lagos Lagos Nigeria; ^2^ Department of Public Health Federal University of Technology Owerri Owerri Nigeria; ^3^ School of Public Policy and Urban Affairs Northeastern University Boston Massachusetts USA; ^4^ Department of Global Health and Development London School of Hygiene and Tropical Medicine London UK; ^5^ Faculty of Management and Development Studies University of the Philippines Open University Los Baños Laguna Philippines; ^6^ Faculty of Public Health Mahidol University Bangkok Thailand

**Keywords:** Africa, health policy, health systems, plastic pollution, treaty, zero plastics

## Abstract

The new plastic treaty, slated for 2024, represents a shift in the global fight to address environmental pollution and degradation. The international agreement, unlike previous resolutions, covers the lifecycle of plastics and represents a legally binding instrument for ending plastic pollution across all ecosystems. Plastic waste contamination poses significant challenges to African nations. It challenges food security, ecological variation and economic development. The African region has been identified with a high level of enactment of waste management policies but a deficiency of sustainable measures to adopt and implement these policies. The new treaty could provide an instrument for collaboration and innovation and set the stage for Africa to transition to a sustainable plastics environment that promotes zero‐waste.

## INTRODUCTION

March 2, 2022, marks an important and unprecedented landmark in the global fight to address global pollution and environmental degradation. At the UN Environment Assembly (UNEA‐5) held in Nairobi, Kenya, UN Member States supported a historic resolution to end plastic pollution and create an international treaty by 2024 [1]. Two small states, Rwanda and Peru, championed the resolution entitled “End Plastic Pollution: Towards an internationally legally binding instrument” that calls for addressing all aspects of plastic's lifecycle, including its creation, use and disposal, and that encompasses all ecosystems [1]. The resolution, built upon three earlier drafts from different countries, helped create an Intergovernmental Negotiating Committee (INC), which would begin work in 2022 and aim to finish a draft of an international agreement by the end of 2024 [2].

The 2022 resolution focuses on the entire life cycle of plastics within and outside any environmental medium [2]. Plastic pollution is increasing worldwide, with plastic waste production accounting for almost 400 million tonnes annually [3]. Only 12% of the plastics manufactured are burned, and only 9% are recycled [3]. The rest is either dumped in landfills or released into the environment, including the oceans. It is estimated that plastics close to the ocean shorelines will exceed 3.5 million tonnes by 2050, more than double the 2020 accumulation [4]

Although plastic pollution in the ocean has received much attention, most UNEP resolutions for addressing plastic pollution have focused on marine plastic [1]. Plastic is also becoming increasingly dangerous to freshwater ecosystems, adversely affecting biodiversity as various aquatic life forms have consumed plastics on purpose or by accident [5]. Microplastics have also been detected in the human placenta and blood [6]. Microplastics have deleterious effects on human health, including male reproduction and sperm quality, making them a possible threat to conception [7].

Plastic pollution is prevalent across Africa, with plastic output and per capita consumption having risen over the past few decades [8]. Population growth, increasing urbanization, inadequate facilities for waste management and lax regulation of municipal waste have resulted in improper disposal of plastic waste and a growing public health risk [8]. However, the growing youthful population is more aware of environmental concerns and open to embracing new technologies, making Africa “especially suitable as a testbed to investigate the effectiveness of new technologies for solving environmental problems” [9]. This commentary examines Africa's leadership in promoting a new UN treaty on plastic pollution, discusses the treaty's potential impact and proposes strategic actions for implementing the treaty.

## THE PROBLEM OF PLASTIC WASTE POLLUTION IN AFRICA

Over the past few decades, plastic production and consumption in Africa have increased significantly for various reasons, leading to a high level of plastic waste contamination. As of 2018, Africa produces 5% and consumes 4% of global plastics volumes, with lax regulation and improving economies contributing to the rising pollution [10]. Most African countries have inadequate recycling capacities, which hinders proper waste management [9]. The limited supply of quality plastic waste input, high cost in the recycling sector and lack of secondary markets for recycled plastics limit effective plastic waste treatment and management [10].

In addition to being an environmental and health concern, plastic waste poses a significant socio‐economic development challenge for African nations. The pollution of rivers, lakes and dams with plastic waste and microplastics endangers irrigation water availability, leading to food insecurity, especially affecting communities that rely on subsistence farming [11]. Urban areas contribute significantly to the growing plastic waste problem by packaging drinking water in single‐use bottles, whereas sachet water production in Africa also significantly contributes to plastic waste accumulation [11, 12]. In 2018, Lagos residents produced 12,000 t of waste per day, with 15% being plastic [10]. Rural towns, burgeoning cities, rivers and coastlines are increasingly polluted with discarded plastic packaging and other plastic waste [13]. These numbers are only growing.

Microplastic pollution is also a significant concern in African water systems, leading to threats of ecological viability when combined with other stressors like climate change [11]. Despite the significant negative impact on various ecosystems, public policy on curbing plastic pollution in Africa is largely inadequate. The high importation of plastics to Africa exacerbates the transboundary nature of plastic pollution [10]. The largest economies in Africa, such as Egypt, Nigeria and South Africa, are also the highest producers and importers of plastics. A total of 33 African countries imported approximately 86.14 Mt of polymers in primary form and 31.5 Mt of plastic products between 1990 and 2017 [13]. As a result, Africa has significantly contributed to the global plastic problem.

## PLASTIC REDUCTION EFFORTS IN AFRICA

Many African countries have made good strides in reducing their plastic waste. Africa has the highest percentage of countries with plastic bag bans (about 46%), with at least 50 African counties having some waste management policies [9]. Rwanda enacted a law banning plastic bags in 2008, which forbids the production, use, import and sale of non‐biodegradable bags that fail to meet its sustainability standards. [9]. Violations are subject to steep fines or imprisonment. In 2017, Kenya also implemented a plastic bag ban, and violation is punishable by fines and jail time though enforcement has been an issue [9]. In May 2019, the Nigerian government enacted the Plastic Bags Prohibition Bill to outlaw the usage, production and importation of all plastic bags used for retail packaging and residential use [12]. However, the effectiveness of these policies could be better as problems persist and plastic waste continues affecting roads, rivers and sewage systems [8]. Drainage systems are choked with plastic bottles, leading to overflow during the rainy season, floods and loss of life.

The Regional Seas Conventions – Kuwait Convention, Barcelona Convention, Basel Convention, Abidjan Convention, Jeddah Convention and Nairobi Convention – are essential in promoting regional cooperation and coordination among nations sharing common resources along their coastlines [14]. They have served as a ground for expressing common regional priorities and establishing regional and global consensus for policy development on marine waste management. The conventions have ensured funding and technical support for parties, the review of regional seas protocols and the adoption and enforcement of regional provisions and commitments.

## THE UN 2022 PLASTIC TREATY AND WHAT IT ENTAILS FOR AFRICA

The UN 2022 Plastic Treaty presents an opportunity for African countries to collaborate and address plastic pollution effectively. Before this resolution, African countries had initiated and adopted policies to reduce plastic pollution, but progress has been limited due to a lack of national commitment and strategic plans [15]. Such policies have stalled, contributing to policy perception prioritization failures. The fragmented nature of initiatives to address various aspects of plastic pollution at national, sub‐regional, regional and global levels has produced insufficient actions [10]. The treaty establishes a framework for international collaboration in addressing plastic waste pollution. The Rwandan government notes that international collaboration is crucial for development [1]. It is essential to create conditions that this treaty will end plastic pollution in Africa.

Studies have shown that most efforts to curb plastic pollution have been at the national and regional levels. Differences in financial capacity, lack of legislative tools, inadequate infrastructure and paucity of binding agreements have limited the effectiveness of measures to reduce plastic pollution [16]. The new treaty could provide an instrument for collaboration and innovation and set the stage for Africa to transition to a sustainable plastic environment that promotes zero‐waste. The resolution's focus on the design of reusable and recyclable products and materials could also help to promote suitable development in Africa. Improved waste management systems, investment in research, mobilization of regional financing for plastic management with a focus on local context and inspiring behavioural change in the African market will ensure that African interventions are adequate, efficient and sustainable.

## STRATEGIC ACTION FOR IMPLEMENTING THE PLASTIC TREATY IN AFRICA

With Rwanda being one of the two leaders of the negotiations on the resolution to end plastic pollution and Senegal hosting the first informal negotiations, Africa has exhibited leadership and commitment. African countries should capitalize on the opportunities presented by the treaty to eliminate plastic pollution on the continent. This treaty could set the pace for regional policy development and collaboration. Policies must reflect the continent's social and cultural diversities based on local contexts.

To achieve the treaty's objectives, African countries should prioritize developing and implementing national strategic plans that address all phases of the plastic lifecycle, with the ultimate goal of zero plastic. The policy framework should adopt circular economy principles and incorporate a shared vision for the plastics system. It should prescribe standards for action for eliminating waste, keeping products and materials in use and regenerating natural systems. The regulatory system must be strengthened with the capacity for enforcement and compliance to achieve policy targets.

Efforts to reduce plastic pollution should focus on hotspots, such as packaging, textiles and the marine sectors, and target collaborative efforts should address hotspots such as drinking water containers. To achieve effective waste reduction, implementation and sustainability, the government should develop and drive plans that work for Africa with support from the UN and development partners. Financing is critical for success, and regional financing should focus on local contexts and prioritize sustainable waste management systems. Trade policies should accelerate the transition to a circular economy for plastics.

Innovation in handling plastic pollution is crucial. African countries should harness the strength and commitment of their young population, who have already shown their capacity for driving change through initiatives and novel ideas while harnessing the expanding power of technology. African youth's voices and actions have been heard and seen in their efforts towards achieving the UN sustainable development goals. Africa is especially suitable as a test bed to investigate the effectiveness of new technologies for solving environmental problems. Promoting sustainable plastics management practices, investing in research and providing access to technology and capacity building are essential motivators.

## CONCLUSION

The problem of plastic pollution in Africa needs serious attention. The evidence that exposure to plastics can impact health negatively shows that plastic pollution is a significant threat to human life. All efforts made by African countries, such as bans on plastic bags, have yet to meet their objectives. The plastic treaty declared by the UN on March 2, 2022, is a step in the right direction towards ending plastic pollution globally. Africans can benefit from the collaboration and innovative opportunities this treaty will offer and reduce plastic pollution on the continent.

## AUTHOR CONTRIBUTIONS

*Writing of draft; editing and review; proofreading*: Deborah Oluwaseun Shomuyiwa. *Writing of first draft; editing; proofreading*: Francisca Ogochukwu Onukansi. *Conceptualization; editing and review; proofreading*: Don Eliseo Lucero‐Prisno III. *Editing; review and proofreading*: Maria Ivanova.

## CONFLICT OF INTEREST STATEMENT

Deborah Oluwaseun Shomuyiwa is a Youth Editorial Board member, and Don Eliseo Lucero‐Prisno III is the Editor‐in‐Chief of Public Health Challenges. They are co‐authors of this article. To minimize bias, they have been excluded from all editorial decision‐making related to the acceptance of this article for publication.

## FUNDING INFORMATION

The authors have received no funding for the development of this paper.

## ETHICS STATEMENT

This is a commentay. There is no need for ethical approval.

## Data Availability

No database or primary data was used in preparing the manuscript.
